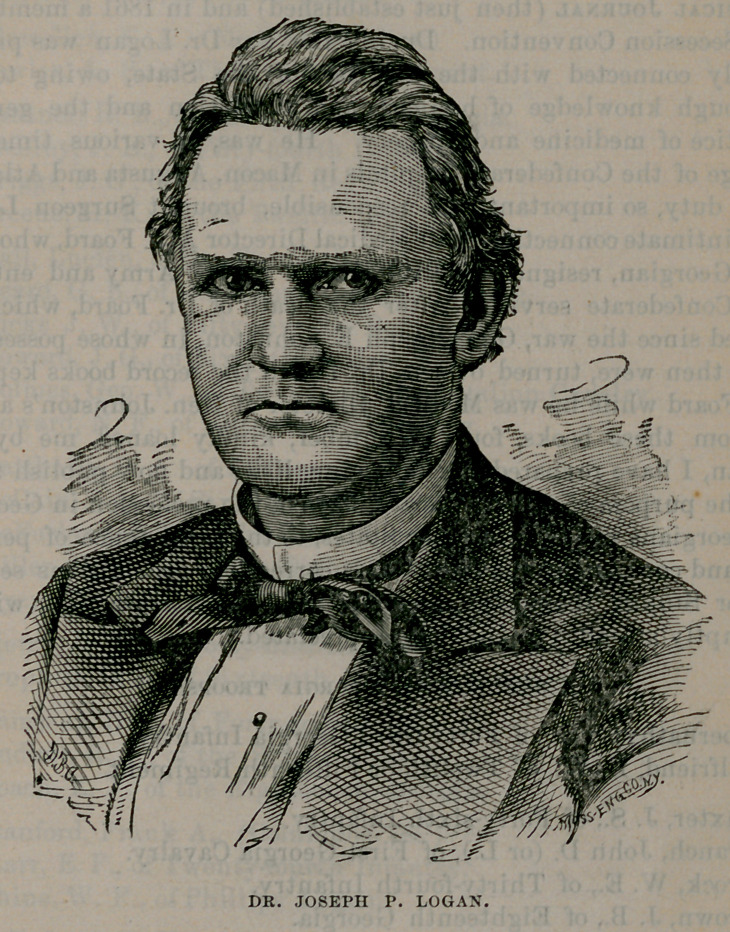# Confederate Surgeons

**Published:** 1884-09

**Authors:** Sidney Herbert

**Affiliations:** Atlanta, Ga.


### CONFEDERATE SURGEONS.

### ROSTER OF SURGEONS WITH GEORGIA TROOPS. HOSPITALS AND THEIR OFFICIALS.

### ARTICLE FIRST—FIELD SURGEONS.

BY SIDNEY HERBERT.

In my endeavor to secure reliable data for a "MILITARY RECORD OF GEORGIA," dating from 1733 to 1883, I have found that the Medical Profession has taken no small part in the wars and conflicts which have marked the past century and a half of the existence of this proud old Commonwealth. During the days of Oglethorpe, and through the later Indian struggles that ended only when the Red Man folded his arms in peaceful submission to fate, and with the "Star of Empire," wended his way "Westward," the Surgeons of the Colony and the State were noted for their heroic courage on the field as well as their skill and fidelity in caring for the sick and wounded.

The same is true of the Mexican war and the "War Between the States," in both of which struggles the Surgeons from Georgia won an enduring fame. It is my purpose, therefore, to set apart a chapter in the "RECORD" to the Surgeons of Georgia, both in the Regular Army and the Volunteer Forces, from the landing of General Oglethorpe down to the present time. I find, however, but little official or historical data from which to compile a reliable and complete list of those Surgeons, and am in consequence compelled to appeal to their survivors, relatives and friends to furnish me such

DR. JOSEPH P. LOGAN.

facts as may be of interest. From this information I can condense a brief but comprehensive record of their services, which will be published in THE JOURNAL as soon as it can be prepared.

There is also great trouble in securing information in regard to living Surgeons who served in the late war, as the State has failed to preserve in a proper manner, her military rosters. By hard work

I have succeeded in compiling an imperfect and partial list of Georgia Surgeons, which is now printed in this connection for the purpose of correction and enlargement. For much of the material I am indebted to Dr. Joseph P. Logan, a distinguished son of Virginia, but for many years past an eminent physician and honored citizen of Atlanta, being in 1856 editor of the ATLANTA MEDICAL AND SURGICAL JOURNAL (then just established) and in 1861 a member of the Secession Convention. During the war Dr. Logan was prominently connected with the hospitals of the State, owing to his thorough knowledge of hygiene and sanitation and the general practice of medicine and surgery. He was, at various times, in charge of the Confederate hospitals in Macon, Augusta and Atlanta. This duty, so important and responsible, brought Surgeon Logan into intimate connection with Medical Director A. J. Foard, who had, as a Georgian, resigned from the United States Army and entered the Confederate service. After the death of Dr. Foard, which occurred since the war, Gen Joseph E. Johnston, in whose possession they then were, turned over to Dr. Logan the record books kept by Dr. Foard while he was Medical Director of Gen. Johnston's army.

From these books, four in number, kindly loaned me by Dr. Logan, I have prepared the following lists, and now publish them for the purpose of calling the attention of the profession in Georgia, or Georgians residing in other States, to the importance of perfecting and completing the lists. Any corrections or additions sent to Major SIDNEY HERBERT, post-office box 36, Atlanta, Ga., will be promptly attended to and duly appreciated.

### SURGEONS OF GEORGIA TROOPS.

Abernathy, J. C., of Forty-third Georgia Infantry.
Allfriend, E. W., of Twelfth or Fifteenth Regiment.

Baxter, J. S., of Forty-sixth Infantry.
Branch, John D. (or L.), of First Georgia Cavalry.
Brock, W. E., of Thirty-fourth Infantry.
Brown, J. B., of Eighteenth Georgia.
Banks, ——, of Thirteenth Infantry.

Carter, J. A., of First Georgia State Line Troops.
Clower, W. P., of Twenty-ninth Regiment.
Cox, H. S., of Forty-second Infantry.
Capeheart, W. R., of First Georgia Confederate.
Calloway, J. J., of Thirty-seventh Regiment

Clark, Chas. E., of Thirteenth(?) Georgia.
Colzey, E. F., of the Third Cavalry.

Davis, J., of Fifteenth Infantry.
Douglas, P. W., of Second State Line Troops.
Davis, N. L., of Twenty-fifth Georgia.

Eldridge, E. J., of Cobb's Legion.

Flewellen, E. A., (Medical Director.)
Fowler, A. S., of Thirty-ninth Regiment.

Gardner, R. B., of Thirty seventh Georgia.
Godfrey, J. E., of Fifty-fourth Infantry.
Griggs, J. G, of the Fifth Regiment.
Greene, H. K., of the Twelfth Georgia.

Hall, Lucien, of the Thirtieth Infantry.
Heard, G. B., of the Second Georgia Cavalry.
Hicks J. W., of Fifty-seventh Regiment.
Howard, J. G., of Sixty-third Georgia.
Holmes, Geo. W., of Twenty-ninth Battalion Cavalry.
Howard, N. F., of Fifty-second Infantry.

Matthews, D. A., of Fifty-seventh Georgia.
McCain, J. S., of Sixty-sixth Infantry.
McFarland, J. T., of the Fifth Cavalry.
Miller, H V. M., of the Eighth Infantry.
Myers, Robert P., of the Sixteenth Georgia.

Pierce, J. W., of Forty-first Infantry.
Prophitt, O. S., of Sixteenth Battalion Cavalry.

Ramseur, D P., of Forty-second Infantry.
Rudicil, R. Y, of the Sixth Cavalry.
Roach, E. J., of the Eighteenth Georgia.

Stanford, Frank A., (Columbus, Ga.) (?)
Starr, E. F., of Twenty-fourth Infantry.
Shine, W. F., of Phillips' Legion.

Taliaferro, V. H., of Second Georgia Battalion.
Thompson, C. R., of the First Infantry.
Terry, Carlisle, (Columbus, Ga.) (?)

Westmoreland, W. F., (Medical Director.)
Watts, E. M., of Fifty-first Georgia.
White, Samuel, of Sixteenth Infantry.

ASSISTANT SURGEONS.

Alexander, M. B., of Forty-third Georgia.
Abernathy, Jones C., of Forty-second Infantry.
Airey, J. D., of Twenty-ninth Georgia.

Broyles, J. J., of the Eighteenth Georgia.
Bringle, W. D., of Wofford's Brigade, Sharpshooters.
Bailey, J. W., Second Georgia State Line Troops.
Bryan, J. H., of Forty sixth Infantry.
Boyd, W. H., of First Georgia Cavalry.
Blackburn, Cary B., of First Georgia Confederate.

Child, J. T., of Fifty-ninth Georgia.
Calloway, J. J., of Ninth Battalion.
Carmichael, W. L., of Third Battalion.
Calhoun, J. E., of Thirtieth Regiment.
Cotton, John F., of Tenth Infantry.
Clements, J. P., of Eleventh Georgia.
Connally, D. H, of Fourteenth Regiment.
Clifton, J. B., of Sixteenth Georgia.

Dickens, S. R., of Thirty-ninth Infantry.

Elliott, W. H., of First Georgia Infantry.
Edelin, J. B., of Fourth Georgia Cavalry.

Farrell, J. W., of the Sixth Cavalry.
Foard, C. F., of First Battalion Sharpshooters.
Franklin, Joel W., of Fifty-sixth Infantry.
Field, S. W., of Phillips' Legion.

Gage, James B., of Thirty-ninth Georgia
Galloway, N. L., of Forty-second Infantry.
Gordon, C. P., of Thirty-fourth Georgia.
Griffin, G. G., of Forty-second Regiment.
Greene, Fred., of the Thirteenth Georgia.

Harden, W. H., of Sixty-fifth Infantry.
Herring, W. E., of Forty-first Georgia.
Harris, R. B., of Fifty-seventh Regiment.
Holcomb, B. W., of Thirty-sixth Georgia.
Hodnett, ——, of the Twelfth Infantry.
Harpe, M. R., of Fifty-second Georgia.
Henry, ——, of Tenth Georgia Cavalry.

Jarrett, A. L., of Forty-seventh Georgia.

King, G. S., of the Fortieth Infantry.
Knott, J. J., of Fifty-third Georgia.

Layton, T. M., of Eighth Georgia Battalion.
Lide, W. R., of Second Battalion Sharpshooters.
Lipford, A. T., of Forty-seventh Infantry.
Lott, E. B., of the Fortieth Georgia.

Matthews, Albert C., of 12th or 15th Georgia. (?)
Mayson, A. S., of the Seventh Infantry.
McManor, of Wofford's Brigade Sharpshooters.
Marlow, N. P., of the Fifth Cavalry.
Melton, John T., of Forty-fifth Regiment.
Morgan, N. A., of the Fifth Georgia.
Myers, R. D., of the Sixteenth Infantry.
Mitchell, T. K., of Twenty-fourth Georgia.
Mu(or o)lkey, H. E., of the Third Georgia Cavalry.
Morris, James, of Second Battalion Infantry.

Oakman, R. H., of Sixty-sixth Regiment.

Parker, John T., of the Eighth Georgia.
Paremore, (?), H. S., of Fiftieth Infantry.
Plunkett, J. D., of the Fortieth Georgia.
Pugh, T. C., of Ninth Infantry Regiment.
Piggatt, W. N., of Wofford's Brigade Sharpshooters.

Ravenel, Edmond, of Twenty-sixth Battalion.
Richardson, M., of Forty-second Infantry.

Sales, J. M., of Twenty-ninth Georgia.
Smith, S. H., of the Tenth Infantry.
Sutherland, A. J., of Second Georgia Cavalry.
Smith, J. N., of the Fourth Cavalry.
Shell, E. C., of Cobb's Georgia Legion.

Tigner, L. H., of Forty-first Infantry.
Treadwell, J. C., of Fourth Battalion Sharpshooters.
Thomas, George, of the Twelfth Georgia.

Wamock (?), R. A., of Fifty-first Infantry.
Wilson, J. A., of Forty-second Georgia.
Wilkerson, T. H., of the Thirtieth Infantry.
Williams, Green B., of Second Georgia Cavalry.
Willis, G. M., of Phillips' Georgia Legion.

Native Georgians residing in other States who have in any way served the military in time of war, either as Surgeons or Assistant surgeons, or Hospital Attendants, are desired to communicate with the writer, and information from any source, if it be reliable, will be thankfully received and duly appreciated. The next article of this series will be on "GEORGIA CONFEDERATE HOSPITALS," about which additional facts, if promptly sent in, might be given, as the record is both incomplete and faulty in many particulars.

*Ellen Villa, Schmidt Station*, 1884.

## Figures and Tables

**Figure f1:**